# Reproductive Capabilities of Female Nilgai (*Boselaphus tragocamelus*) in Southern Texas

**DOI:** 10.3390/ani14152150

**Published:** 2024-07-24

**Authors:** Megan M. Granger, Clayton D. Hilton, Scott E. Henke, Humberto L. Perotto-Baldivieso, Landon R. Schofield, Tyler A. Campbell

**Affiliations:** 1 Texas Parks and Wildlife Department, 2400 Smith School Road, Austin, TX 78744, USA; megan.granger@tpwd.texas.gov; 2 Department of Rangeland and Wildlife Sciences, Texas A&M University-Kingsville, 700 University Boulevard, Kingsville, TX 78363, USA; clayton.hilton@tamuk.edu (C.D.H.); scott.henke@tamuk.edu (S.E.H.); 3 Department of Rangeland, Wildlife and Fisheries Management, Texas A&M University, 2138 TAMU, 495 Horticulture Road, College Station, TX 77843, USA; humberto.perotto@ag.tamu.edu; 4 East Foundation, 200 Concord Plaza Drive, Suite 410, San Antonio, TX 78216, USA; lschofield@eastfoundation.net

**Keywords:** nilgai, pregnancy rates, reproduction, twinning

## Abstract

**Simple Summary:**

To better inform nilgai antelope management decisions, basic life history data are needed. We collected reproductive tracts of free-ranging female nilgai from 2018 to 2021 in Southern Texas. We found high pregnancy and twinning rates. Moreover, we found nilgai as young as 1 year old to be pregnant and nilgai as old as 12 years old to be pregnant. Our findings demonstrate the high reproductive potential of free-ranging nilgai in Southern Texas. To prevent nilgai overpopulation and associated damage to the land, harvest management strategies should be used, particularly in introduced areas where there are no natural predators.

**Abstract:**

Free-ranging nilgai antelope (*Boselaphus tragocamelus*) are an understudied species, both on their native ranges of India, Pakistan, and Nepal and on their introduced ranges in southern Texas. Basic data related to population sizes, survival, reproduction, and recruitment are needed throughout their range to inform management and conservation decisions. We collected nilgai fetuses from 3 ranches in southern Texas, including East Foundation’s El Sauz and Santa Rosa ranches, and the Norias Division of the King Ranch^®^ from 2018–2021. We calculated the percentage of individuals that were pregnant in each of the sample years and overall. We determined monthly average, maximum, and minimum fetus length. Of 488 nilgai cows, we found 386 to be pregnant (79%) and 214 to be pregnant with twins (56%). We found nilgai cows as young as 1-year old to have fetuses and therefore to have reached sexual maturity. Sex ratios of fetuses during any sampling year did not differ. We found ample evidence supporting our hypothesis that nilgai are fecund on their introduced range of southern Texas. To prevent nilgai overpopulation and associated problems, harvest management strategies should be implemented, specifically on nilgai cows.

## 1. Introduction

Free-ranging nilgai antelope (*Boselaphus tragocamelus*) are an understudied species, both in their native ranges of India, Pakistan, and Nepal and in their introduced ranges in Southern Texas. Basic data related to population sizes, survival, reproduction, and recruitment are needed throughout their range in inform management and conservation decisions. Factors impacting nilgai abundance and management are different in their native and introduced ranges. For example, nilgai in their native ranges are limited by poaching, habitat deterioration, and predation by tigers (*Panthera tigris tigris*) [1,2], whereas nilgai in their introduced ranges in Texas are limited by legal harvests and prolonged periods (>48 h) of below-freezing temperatures [3], with no known predators [4].

Though highly restricted by scope and sample size, prior studies of nilgai in their introduced ranges of Texas have demonstrated (1) high reproduction rates in captive environments, (2) female nilgai reaching sexual maturity at 2 years old in free-ranging environments [4], and (3) that twinning is common in free-ranging environments (~50%) [5].

The East Foundation began implementing its Exotic Ungulate Control Project in 2013. The aim of this project is to reduce the damage to the land caused by wild pigs (*Sus scrofa*) and nilgai. Between 150 and 300 cow or juvenile male nilgai are removed annually. This is accomplished through commercial meat harvests from a helicopter by a wild meat vendor (www.brokenarrowranch.com accessed on 20 June 2024). Following harvests, carcasses are prepared for cold shipping onsite. Inedible portions of the carcass (offal, skins, etc.) are recycled on ranches. This provided us with the opportunity to collect, record, and compare data from the reproductive tracts of free-ranging nilgai cows and—most importantly for our purposes here—nilgai fetuses.

Our objectives were to evaluate free-ranging nilgai cow reproduction metrics, including the (1) age of sexual maturity as determined by the presence of a fetus, (2) pregnancy rates, (3) twinning rates, (4) fetal growth rates, and (5) fetal sex ratio. Consistent with the limited findings of others, we hypothesized that free-ranging nilgai cows in their introduced ranges in Southern Texas would be highly fecund.

## 2. Materials and Methods

### 2.1. Study Area

**Figure 1:**
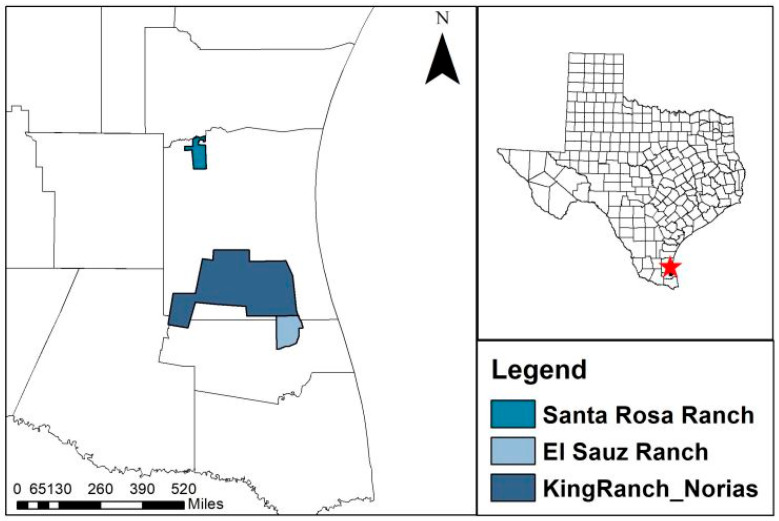
Red star is location of East Foundation’s Santa Rosa and El Sauz ranches and King Ranch^®^ Norias Division, where we collected fetuses from pregnant nilgai (*Boselaphus tragocamelus*) cows from 2018 to 2021.

We collected nilgai fetuses from 3 ranches in Southern Texas (Figure 1), including the East Foundation’s El Sauz and Santa Rosa ranches and the Norias Division of the King Ranch^®^ (Kingsville, TX, USA). These ranches occur within 3 ecoregions, including the South Texas Brush Country, the Coastal Prairies and Marshes, and the South Texas Sand Sheet [6]. Common vegetation that occurs throughout these ranches is live oak (*Quercus virginiana*), honey mesquite (*Prosopos glandulosa*), huisache (*Acacia farnesiana*), lime prickly ash (*Xanthoxylum americanum*), granjeno (*Celtis pallida*), brasil (*Condalia hookeri*), gulf cordgrass (*Spartina spartinae*), and seacoast bluestem (*Schizachyrium scoparium*) [7].

### 2.2. Sample Collection

Broken Arrow Ranch (www.brokenarrowranch.com accessed on 20 June 2024) performed aerial meat harvests of female and juvenile male nilgai in May–July 2018 (*n* = 130), April–June and August 2019 (*n* = 141), May–July and September 2020 (*n* = 210), and April–May 2021 (*n* = 118) across our 3 study sites. We placed each harvested animal in a nilgai age class based on the cementum annuli and tooth eruption and wear patterns [8]. Specifically, we identified female nilgai < 1 year old as class 1, 1 year old as class 2, 2–4 years old as class 3, 5–7 years old as class 4, 8–11 years old as class 5, and >11 years old as class 6 [8]. We assessed the lactation status of each cow through milk expression from the teats. We assessed the pregnancy status of each cow by the presence of a fetus in the amniotic sac. If pregnant, we recorded the number of fetuses per cow. We determined the sex of fetuses, when possible, and we recorded the crown rump length in centimeters.

### 2.3. Data Analyses

We calculated the percentage of individuals that were pregnant in each of the sample years and overall. Next, using only the pregnant cows, we calculated the percentage of those pregnant cows that were carrying twins in each year. We separated the cows into age classes: class 1 = juveniles (<1 year old), class 2 = 1 year old, class 3 = 2–4 years old, class 4 = 5–7 years old, class 5 = 8–11 years old, and class 6 = ≥12 years old [8]. We used Kruskal–Wallis tests to compare the means of (1) the percentage of cows that were pregnant and (2) the percentage of pregnancies that carried twins among age classes 2–5 across all four years. Age class 1 was not included in these analyses because there were no pregnant individuals in this age category.

We determined the monthly average, maximum, and minimum fetus length. We conducted chi-square analyses to compare the number of female and male fetuses in each year of collection. We conducted all statistical analyses using SPSS version 27.0 [9]. We considered statistical significance at *p* < 0.05.

## 3. Results

**Figure 2:**
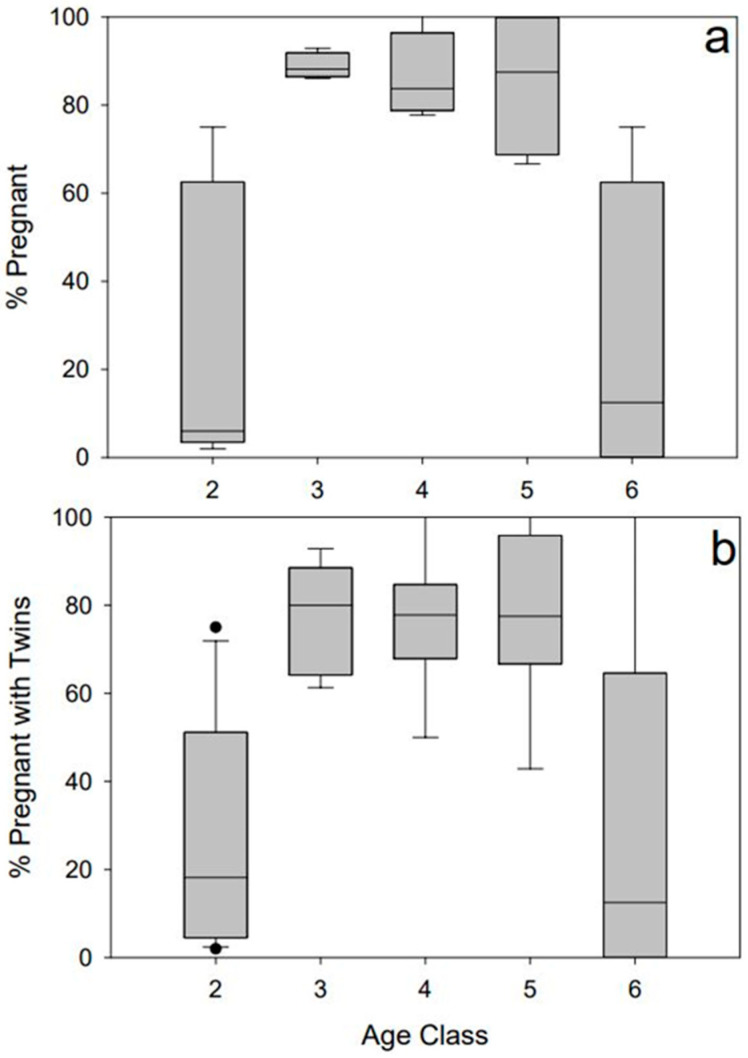
(**a**) Percentages of pregnant nilgai (*Boselaphus tragocamelus*) cows that were sampled on 3 Southern Texas ranches from 2018 to 2021 by age class (class 2 = 1 year old, class 3 = 2–4 years old, class 4 = 5–7 years old, class 5 = 8–11 years old, and class 6 = ≥12 years old [8]). (**b**) Percentages of nilgai pregnancies that were twins by age class (see above) on 3 Southern Texas ranches from 2018 to 2021. We considered statistical significance at *p* < 0.05.

We sampled 599 total nilgai, of which 488 were females of reproducing age (class 2 ≥ 1 year old). Of these 488 nilgai cows, we found 386 to be pregnant (79%) and 214 to be pregnant with twins (56%) (Table 1). No differences were observed between the years for the percentage of pregnant females (*χ*^2^ = 1.61, *p* = 0.66) or for the percentage of pregnancies that carried twins (*χ*^2^ = 4.4, *p* = 0.22). The percentages of cows that were pregnant differed by age class (H = 12.283, *p* = 0.015; Figure 2a), with age classes 3–5 (ages 2–11 years old) demonstrating the highest pregnancy rates (67–100% pregnant). The percentages of pregnancies that carried twins did not differ by age class (H = 8.319, *p* = 0.081; Figure 2b), although there was a trend for classes 3–5 to be more likely to carry twins than younger (class 2) and older (class 6) cows. We found that nilgai cows as young as 1 year old (i.e., age class 2) reached sexual maturity. Although we cannot determine at what age nilgai cows are no longer capable of reproducing, the oldest cementum-annuli-aged nilgai that was pregnant in this study was 12 years old [7].

**Figure 3:**
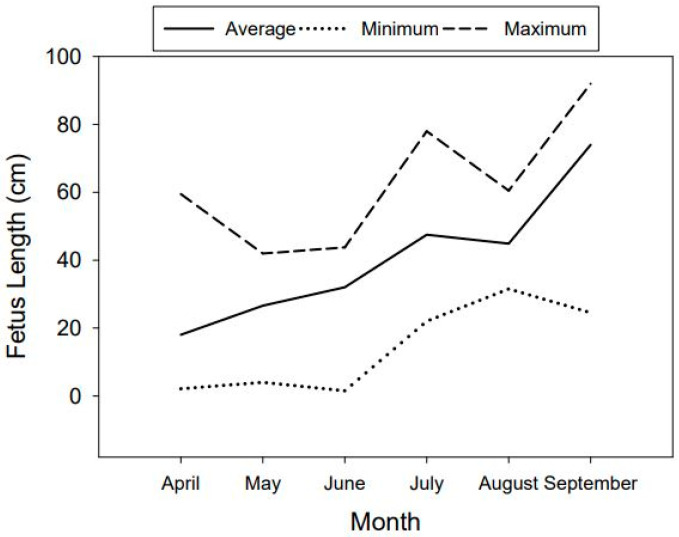
Average, maximum, and minimum fetus crown rump lengths of fetuses collected from pregnant nilgai (*Boselaphus tragocamelus*) cows (≥1 year old) by month, which were sampled on 3 Southern Texas ranches from 2018 to 2021. The solid line is the average length, the dotted line is the minimum length, and the dashed line is the maximum length.

We collected a total of 636 fetuses after harvests across all four years. Beginning in April, the average fetus length was 18.04 cm, and this average steadily increased with each successive month until September, when the average length was 73.98 cm (Figure 3). During September (the latest month of the year of our seasonal harvests), there were four pregnant females that were lactating and had a fetus(es) that were over 78 cm in length, with the largest measuring 92 cm. We have observed that crown rump fetus lengths of 78 cm are close to term. This indicates that the nilgai peak calving season begins in mid-September. The sex ratios of fetuses during any sampling year did not differ (Table 2).

## 4. Discussion

We found ample evidence supporting our hypothesis that nilgai are fecund in their introduced range of Southern Texas. Specifically, nilgai had high pregnancy and twinning rates. Additionally, we found that cows were capable of reproducing when as young as 1 year old. Ungulates with large body sizes and rapid rates of body growth are typically able to begin reproducing at earlier ages than species with smaller body sizes [10,11]. Nilgai cows fit into the large body size category, typically weighing between 100 and 213 kg [4], which explains how they can reproduce at a young age. Like other keystone herbivore species, nilgai are reproductively mature at young ages. Our oldest nilgai cow that was pregnant was 12 years old, based on cementum annuli analyses [8]. These data demonstrate that the reproductive lifespan of nilgai cows in Southern Texas is at least 11 years but could be longer. Although there were several individuals in age class 6 (ages ≥ 12 years) that were pregnant, further research and a larger sample size would be necessary to determine at what age reproductive senescence occurs. Lastly, our results showed higher pregnancy and twinning rates than prior captive studies [4].

The average fetal crown rump lengths by month showed the longest fetal lengths in September. Based on the maximum and average length, and an increase in positive lactation statuses in individuals at the beginning of September, we surmise that their peak calving season begins in mid-September and continues into early November. Considering that nilgai have an average gestation period of 245 days [12], the beginning of the peak breeding season would start in mid-January and continue into early March. However, the high variation in the maximum and minimum values of the fetal crown rump lengths and the number of adult nilgai cows that were lactating and not pregnant (Table 1) showed that nilgai are capable of breeding throughout the year. A 1:1 fetal sex ratio is common and expected in most mammalian species because natural populations have strong frequency-dependent selection in the even production of males and females [13]. Nilgai were consistent with this and presented an even fetal sex ratio.

## 5. Conclusions

Our results show that nilgai are highly capable of increasing their population abundance in Southern Texas if harvest management efforts are not implemented. For example, assuming average pregnancy and twinning rates, each cow nilgai would produce >13.5 calves over the course of an 11-year reproductive duration. Nilgai are a management concern because they cause property damage by creating fence breaches (areas of net wire fencing where an animal pushes the fence up to cross under) in livestock fencing [14]. Nilgai are also a major concern because they are known carriers of cattle fever ticks (*Rhipicephalus annulatus* and *R. microplus*) [15], which can transmit babesia organisms (*B. bovis and B. bigemina*) to cattle [16], although nilgai do not appear to be susceptible to the disease [17]. The overpopulation of large ungulate species, such as nilgai, can lead to the degradation of landscapes [18], competition with cattle and white-tailed deer (*Odocoileus virginianus*) for forage [19], and the increased spread of wildlife diseases [20]. To prevent these issues from occurring, harvest management strategies should be implemented, specifically on nilgai cows, to keep nilgai populations under control. These efforts will aid in preventing nilgai populations from continuing to increase unabated.

## Figures and Tables

**Table 1:** Adult (≥1 year old) nilgai (*Boselaphus tragocamelus*) cow pregnancy, twinning, and lactation rates by year from 2018 to 2021 on 3 ranches in Southern Texas.

Year	# Adult Cows	Pregnant		Pregnant with Twins		Lactating	
		*n*	%	*n*	%	*n*	%
2018	114	93	82	60	65	1	<1
2019	124	99	80	50 *	51	8	6
2020	158	125	79	69	55	23	11
2021	92	69	75	35	51	34	30
Overall	488	386	79	214	55	66	14

* One cow was pregnant with triplets in 2019.

**Table 2:** Number of fetuses collected from nilgai (*Boselaphus tragocamelus*) cows (≥1 year old) by year (from 2018 to 2021) and sex on 3 ranches in Southern Texas.

Year	*n*	Sex Ratio		Chi-Square	*p*-Value
		Female	Male		
2018	204	107	97	0.397	0.529
2019	142	81	61	2.540	0.110
2020	202	107	95	0.599	0.439
2021	88	48	40	0.557	0.455
Total	636	343	293	3.780	0.052

## Data Availability

The original contributions presented in the study are included in the article; further inquiries can be directed to the corresponding author.

## References

[1] Khanal S., Aryal A., Morley C.G., Wright W., Singh N.B. (2017). Challenges of conserving blue bull (*Boselaphus tragocamelus*) outside the protected areas of Nepal. Proc. Zool. Soc..

[2] Basak K., Mandal D., Babu S., Kaul R., Ashraf N.V.K., Singh A., Mondal K. (2018). Prey animals of tiger (*Panthera tigris tigris*) in Dudhwa landscape, Terai region, north India. Proc. Zool. Soc..

[3] Goolsby J.A., Saelao P., May M., Goldsmith B. (2021). Nilgai (*Boselaphus tragocamelus*) mortality levels in South Texas after historic freeze event. Subtrop. Agric. Environ..

[4] Mungall E.C., Sheffield W.J. (1994). Exotics on the Range: The Texas Example.

[5] Sheffield W.J. (1983). Food habits of nilgai antelope in Texas. J. Range Manag..

[6] Bailey R.G., Avers P.E., King T., McNab W.H. (1994). Ecoregions and Subregions of the United States (Map; Scale 1:75,000,000).

[7] Fulbright T.E., Diamond D.D., Rappole J., Norwine J. (1990). The Coastal Sand Plain of southern Texas. Rangelands.

[8] Granger M.M., Hilton C.D., Henke S.E., Perotto-Baldivieso H.L., Schofield L.R., Campbell T.A. (2024). Determining the age classes of free-ranging nilgai in southern Texas. Wildl. Soc. Bull..

[9] IBM Corporation (2020). IBM SPSS Statistics for Windows.

[10] Millar J.S. (1977). Adaptive features of mammalian reproduction. Evolution.

[11] Green C.H., Rothstein A. (1991). Trade-offs between growth and reproduction in female bison. Oecologia.

[12] Leslie D.M. (2007). *Boselaphus tragocamelus* (Artiodactyla: Bovidae). Mamm. Species.

[13] Wedekind C., Povilitis T. (2012). Managing Population Sex Ratios in Conservation Practice: How and Why?.

[14] Zoromski L.D., DeYoung R.W., Goolsby J.A., Foley A.M., Ortega-Santos J.A., Hewitt D.G., Campbell T.A. (2022). Animal use of fence crossings in southwestern rangelands. Ecol. Evol..

[15] Cárdenas-Canales E.M., Ortega-Santos J.A., Campbell T.A., García-Vázquez Z., Cantú Covarrubias A., Figueroa-Millán J.V., DeYoung R.W., Hewitt D.G., Bryant F.C. (2011). Nilgai antelope in northern Mexico as a possible carrier for cattle fever ticks and *Babesia bovis* and *Babesia bigemina*. J. Wildl. Dis..

[16] Bock R., Jackson L., De Vos A., Jorgensen W. (2004). Babesiosis of cattle. Parasitology.

[17] Johnson T.L., Persinger K.A., Taus N.S., Davis S.K., Poh K.C., Kappmeyer L.S., Laughery J.M., Capelli-Peixoto J., Lohmeyer K.H., Ueti M.W. (2024). Nilgai antelope display no signs of infection upon experimental challenge with a virulent *Babesia bovis* strain. Parasites Vectors.

[18] Manier D.J., Hobbs N.T. (2007). Large herbivores in sagebrush steppe ecosystems: Livestock and wild ungulates influence structure and function. Oecologia.

[19] Hines S.L., Fulbright T.E., Ortega-Santos J.A., Wester D.B., Hewitt D.G., Boutton T.W., Campbell T.A. (2024). Impacts of large herbivores, abiotic factors, and site production on plant species richness in a semiarid grassland. J. Arid Environ..

[20] Caron A., Miguel E., Gomo C., Makaya P., Pfukenyi D.M., Foggin C., Hove Y., Wichatitsky M.G. (2004). Relationships between burden of infection in ungulate populations and wildlife/livestock interfaces. Epidemiol. Infect..

[21] Sikes R.S., Animal Care and Use Committee of the American Society of Mammalogists (2016). Guidelines of the American Society of Mammalogists for the use of wild mammals in research and education. J. Mamm..

